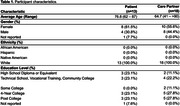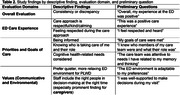# Emergency Department Experiences of People Living with Dementia

**DOI:** 10.1002/alz.084586

**Published:** 2025-01-09

**Authors:** Andrea L Gilmore‐Bykovskyi, Laura M Block

**Affiliations:** ^1^ Wisconsin Alzheimer’s Disease Research Center, Madison, WI USA; ^2^ University of Wisconsin‐Madison School of Nursing, Madison, WI USA; ^3^ University of Wisconsin Madison School of Medicine & Public Health, Madison, WI USA

## Abstract

**Background:**

Over half of the 6 million persons living with dementia (PLWD) in the United States visit emergency departments (EDs) annually. Because EDs play a vital role in providing care to PLWD, there has been increased attention to improving the ED care experience. Yet, measures to evaluate the ED care experience for PLWD focus predominantly on care utilization or distal outcomes (e.g., ED revisit, mortality) and may not adequately address aspects of the ED care experience itself or what matters most to PLWD during ED visits. To address this gap, this study aims to identify care priorities of PLWD during ED visits to inform the development of a Person‐Centered Outcome Measure for use by PLWD to facilitate evaluation of those priorities.

**Methods:**

This presentation will discuss findings from interviews with PLWD and care partners of PLWD during ED encounters which were analyzed using thematic analysis. Descriptive findings were categorized into one of four inductively‐derived evaluation domains, from which preliminarily evaluation questions were developed.

**Results:**

Interviews were conducted with 31 participants, consisting of 15 care partners, 10 PLWD, and 3 dyads (Table 1). Descriptive findings reported by both PLWD and care partners emphasized their priorities for ED care, including the importance of being informed about the care and testing they received, knowing the roles of those on their care teams, and feeling that their goals for care were addressed. Some PLWD and care partners evaluated domains differently; i.e., overall evaluated ED experience positively but goals of care were not met. Table 2 presents the evaluation domains derived through analysis and preliminary questions designed to facilitate evaluation of these domains.

**Conclusions:**

Findings demonstrate that evaluation of ED care experiences is complex and multi‐faceted, and emphasize that assessment strategies are needed that target multiple domains of the experience including goal alignment, approach to care, and overall perception of experience. Findings indicate PLWD and care partners value feeling informed in the ED, and many cite the importance of being aware of the impact of cognition. Evaluations of the ED experience differs between PLWD and care partners, and by stage of dementia, requiring further investigation.